# The challenges of international collaboration in conflict and health research: experience from the Research for Health in Conflict-Middle East and North Africa (R4HC-MENA) partnership

**DOI:** 10.1186/s13031-023-00527-8

**Published:** 2023-06-14

**Authors:** Chiu-Yi Lin, Kristen Meagher, Martin Bricknell, Preeti Patel, Nassim El Achi, Tezer Kutluk, Richard Harding, Hanna Kienzler, Rita Giacaman, Deborah Mukherji, Omar Shamieh, Richard Sullivan

**Affiliations:** 1 Centre for Conflict and Health Research, King’s College London, London, UK; 2 Department of Pediatric Oncology, Hacettepe University Faculty of Medicine and Cancer Institute, Ankara, Turkey; 3 Florence Nightingale Faculty of Nursing Midwifery & Palliative Care, Cicely Saunders Institute, King’s College London, London, UK; 4 Department of Global Health & Social Medicine, School of Global Affairs, King’s College London, London, UK; 5 Institute of Community and Public Health, Birzeit University, West Bank Birzeit, Occupied Palestinian Territory; 6 Department of Internal Medicine, Division of Hematology-Oncology, American University of Beirut Medical Center (AUBMC), Beirut, Lebanon; 7 Center for Palliative & Cancer Care in Conflict, King Hussein Cancer Center, Amman, Jordan; 8 King’s Institute Cancer Policy, King’s College London, London, UK

**Keywords:** International collaboration, R4HC-MENA, Health research in conflict, Capacity strengthening, Global health, Qualitative study

## Abstract

**Background:**

Healthcare is a basic human right extending across all humanitarian contexts, including conflict. Globally, two billion people are living under conditions of insecurity and violent armed conflict with a consequent impact on public health. Health research in conflict-affected regions has been recognised as important to gain more understanding of the actual needs of such populations, to optimise healthcare delivery, as well as to inform advocacy and policy change. International collaborative research maximises the resources and skills available for dealing with global health issues, builds capacity and endeavours to ensure the research reflects real needs of the populations. Under the UK’s Global Challenge Research Fund in 2017 a number of such international programs were created including the Research for Health in Conflict-Middle East and North Africa (R4HC-MENA) partnership to build capacity in conflict and health research as well as study specific areas, namely noncommunicable diseases in conflict (cancer & mental health) and the political economy of health in conflict.

**Methods:**

A qualitative study using semi-structured online interviews was conducted to explore researchers’ and stakeholders’ perspectives on the R4HC-MENA programme over its lifetime from 2017 to 2021. It aimed to understand the factors that influenced and accelerated international collaboration within the R4HC-MENA programme on conflict and health research, and to provide deeper insights into the implementation of the programme. Data collection was conducted from March 2022 to June 2022. Purposive and snowball sampling techniques were used for participant recruitment. Thematic analysis was applied for data analysis.

**Results:**

Twelve researchers/stakeholders participated in this study: four men and eight women. Four main themes were generated: Theme 1: Network building (personal and institutional levels); Theme 2: Hierarchies and power dynamics (power imbalance between different academic status, genders and institutions); Theme 3: Communication challenges; Theme 4: Career development (management, leadership, research, and teaching skills).

**Conclusions:**

This study provided preliminary insights into perspectives on international collaboration in a major international programme of research on conflict and health. Several key challenges and outputs were generated by the researchers in this study. The findings are important for further developing effective strategies to tackle the challenge of power imbalance and ineffective communication in international research collaborations.

## Background

Access to healthcare is a basic human right and minimum standards should apply in humanitarian contexts, including conflict [1]. The Sphere Handbook states: “*People affected by disaster or conflict have the right to life with dignity and, therefore, the right to assistance; and all possible steps should be taken to alleviate human suffering arising out of disaster or conflict*” [1]. However, two billion people are living under conditions of insecurity and vicious armed conflict with a consequent impact on population health [2].

In order to provide evidence-based solutions for this, building a body of research in conflict settings is crucial. Nevertheless, some disagreement on the necessity for conducting research in conflict-affected populations has been raised in the field. Given the vulnerability of the populations, some have argued, there is no need to burden them with participating in research, and the resources for conducting research could be used for more urgent priorities, such as providing life-saving medicines [3, 4].

By contrast, health research in conflict-affected regions has been recognised as an important tool to gain more understanding of the actual health needs of affected populations, to optimise the efficiency of and further improve healthcare delivery, and to inform advocacy and policy change [4, 5]. Furthermore, many commentators have reflected that accurate epidemiological information and access to and improved delivery of relief assistance support social and political changes in conditions of protracted conflict. With these research efforts, tangible benefits to the general public in conflict-affected settings could be expected and could lead to better health outcomes.

It is imperative that prospective researchers and humanitarian workers in global health understand the health implications of emergency and conflict-affected settings [6]. International collaborative research maximises resources and skills towards global issues and helps to ensure that the impact of research programmes is communicated to many countries and stakeholders [7]. There are also mutual benefits of international collaboration for both host and partner countries [8, 9]. More widely such programs challenge and encourage interactions among countries to critique Western dominate frameworks in the field of conflict and health [10]. Such critical examination leads to tangible changes in pedagogy, for example, developing a course on qualitative research methods in mental health and conflict, that is co-developed and situated in the unique context of those with lived experiences [10]. The critical reflections drive two-way learning process for all stakeholders based on equal partnerships [9, 11].

The R4HC-MENA (Research for Health Conflict—Middle East and North Africa) research programme, hosted by the Centre for Conflict and Health Research at King’s College London, is an example of strengthening research and policy capacity relevant to conflict-affected settings in the Middle East and North Africa [12]. The aim of this programme was to strengthen research and policy capacity related to conflict affected areas, focusing on health, political economy of health, complex non-communicable diseases (such as mental health and cancer), and facilitate more effective translation of research into policy. To achieve the aim, a series of contextually and culturally sensitive activities were organised, including co-development and co-delivery of accredited multi-disciplinary courses/training and locally driven research programmes, establishing new sustainable partnerships between organisations/institutions to build expertise and research capacity, and mentoring and supporting senior leaders in the translation of research into policy in the organisations/ institutions. The collaboration, between seven academic institutions, was based on equal partnerships, reciprocal north–south and south-south learning, and building a community of practice. There were four inter-related workstreams: (1) conflict and health (to build regional capacity on conflict and health); (2) the political economy of health in conflict (to provide systematic and empirically grounded research capacity in the political economy of health in conflict in MENA countries); (3) cancer and palliative care (to explore and identify unprecedented challenges of cancer and palliative care in conflict); and (4) mental health research in regions of conflict (to understand training needs assessment for mental health research in conflict). Across all four work streams, UK and MENA partners were equal in co-developing and co-delivering curricula and courses in local contexts, including a wide range of learning technologies and informatics (such as blended learning and virtual learning).

To build academic depth and ensure the translation of research into policy, both UK and MENA partners were encouraged to co-publish in high impact journals. A wide range of research topics have been covered by the group, including COVID-19 [13–18], cancer care [19–23], palliative care [15, 24], gender equity [18, 25], military healthcare [26–29], and mental health care [30–32] in conflict-affected settings (including Syria, Lebanon, Jordan, Palestine, and Turkey). This programme was funded by a £5.9 million grant from the UK Research and Innovation (UKRI) Global Challenges Research Fund and ran from September 2017 to March 2022. Two of the four years of the programme were during the height of the COVID-19 pandemic and therefore several face-to-face capacity strengthening activities had to take place online. This research group published a total of 115 academic papers and reports [33]. In order to ensure efficient communications among stakeholders, 12 Executive Board meetings, primarily in Jordan, Turkey and Lebanon were held. Meanwhile, five conferences were hosted across the network such as R4HC-MENA International Policy Conference on Health in conflict in Cambridge, UK, in 2022 and Research for Health in the Syrian Conflict Conference in 2019 and 2020 (virtual conferences). More than 40 workshops were held across different work streams of the R4HC-MENA programme, such as Cancer Control, Palliative Care, and Mental Health workshops in Turkey; Health Economics, Psychometrics, and Person-Centred Care, in Jordan. Beyond these, significant outcomes from the programme have been identified: 1. Further grants (National Institute for Health and Care Research, NIHR funded £4 m Research for Health Systems Strengthening in Syria (R4HSSS) programme); 2. Developing programmes/courses (COVID-19 and Cancer Global Taskforce; The Women Leaders in Health and Conflict (WLHC) supported by the Foreign, Commonwealth & Development Office (FCDO) and NIHR; The FutureLearn course “Qualitative Research Methods for Mental Health in War and Conflict” collaboratively developed between King’s and Birzeit University; intensive qualitative and quantitative training courses delivered by scholars from King’s and Birzeit at the Institute of Community and Public Health at Birzeit University; The online certificate in conflict medicine at the Global Health Institute – American University of Beirut, Lebanon); and three establishing research centres (such as The Centre for Palliative & Cancer and Conflict at King Hussein Cancer Centre, Jordan).

It is important to explore what can be learnt from the experience. Therefore, this study aimed to explore researchers’ and stakeholders’ perspectives on the international collaborative study, the R4HC-MENA programme.

## Method

### Study design

A qualitative study using 12 semi-structured interviews (online) was conducted from March to June 2022 to explore researchers’ and stakeholders’ perspectives (including Principal Investigator, Co-investigators, and programme /project managers) at the end of the international collaborative research programme, R4HC-MENA. The aim was to understand the factors that influenced and accelerated international collaboration in the R4HC-MENA programme on conflict and health research, and to provide deeper insights into the implementation of the programme. Although the findings of qualitative interviews are not expected to be generalisable [34], the purpose of this study was to provide more in-depth analysis of the experiences of participants in the programme and to share lessons with the world on how to replicate such successful consortia and adapt them for other contexts. Therefore, the participants involved in the R4HC-MENA programme were selected to best enable the researchers to meet the aims of this programme.

### Participants and settings

The key informants were recruited from the R4HC-MENA partnership. The recruitment information was advertised through emails and during the R4HC conferences. Two sampling methods were selected for use: purposive sampling, and snowball sampling. Although potential participants who were working on the projects during the data collection were contacted through the R4HC-MENA network, the snowball sampling technique – where initial participants recruit other possible participants from their social networks – was applied to access other potential participants who had previously worked on projects within the programme. The inclusion criteria were that participants must be researchers and stakeholders (including Principal Investigator, Co-investigators, and programme /project managers) who were involved in the R4HC-MENA programme either formerly or now, and able to communicate in English.

### Data collection

To explore participants’ perspectives, factors that influenced international collaboration, and insights into the implementation of the programme, this study used in-depth semi-structured online interviews, with a topic guide. Potential participants were provided with a participant information sheet about this study by the research team. The interviews were held at times which were mutually convenient for the participants and the research team. All participants needed to sign an informed consent form before data collection. All interviews were digitally recorded and transcribed verbatim by the research team (CL and KM). Field notes were taken during the interviews to provide a context to inform data analysis and facilitate the authors’ reflection at a later stage.

Data collection in this study continued until the data saturation point was reached. The data saturation point was identified as being when no new codes or themes were generated from additional data [35]. After nine interviews, no more new codes or themes were generated, but in order to confirm data saturation, three more interviews were conducted.

### Data analysis

Thematic analysis as proposed by Braun and Clarke was used in this study. There are six steps: (1). familiarisation with the data; step; (2). generating initial codes; (3). searching for themes; (4). reviewing themes; (5). defining and naming themes; (6). producing the report [36]. During the data analysis, NVivo software (Version 12) was used to manage and analyse the data.

Following the completion of the transcription, the lead author (CL) read all the transcripts at least three times to ensure the interviews had gathered the information needed for this study. After this, the author then coded them with an initial code, and a long-list of codes was generated. After coding, the author grouped the codes into initial themes. Finally, the main themes were identified. During the process, the primary authors (CL and KM) held regular meetings and discussions to ensure the themes represented the full dataset.

### Ethical considerations

Ethical approval was obtained from King’s College London Ethics Review Committee (Ref: MRA-21/22–28,673, 01/03/2022). All participants voluntarily took part in the study and signed the informed consent. Participants were informed of their right to withdraw from the study at any time during and immediately after the interview without giving any reason. Participants were not able to withdraw their data after the data was anonymised and analysed as it would not be possible to identify their records. Participants were assured of confidentiality and anonymity, both during the study process and in academic publications. All audio-recording files, consent forms, and transcripts were stored securely. All these matters were explained clearly in the participant information sheet for participants.

### Rigour

In this study, the principles of rigour proposed by Lincoln and Guba were applied to ensure quality, including credibility, transferability, dependability, and confirmability [37]. In order to improve credibility, peer debriefing was used to obtain guidance from the other academic authors. A thick description of the study process, including the aim of the study, and details of data collection, data analysis, and sampling, were provided to enhance transferability. An audit trail was also maintained to document features of the study design to ensure dependability. Reflective journal was carried out to support confirmability in this study.

## Results

The study was conducted between March 2022 and June 2022. Twelve participants who were working or had worked for the R4HC-MENA partnership agreed to be interviewed and then took part in the interviews. Six researchers and six stakeholders participated this study. The participants comprised four men and eight women with an average age of 45 years (Median = 44 years old, three participants did not respond). Eight participants were based in the UK, and four were based in other countries (two in occupied Palestinian territory, one in Jordan, one in Turkey). Interview duration ranged from 20 to 57 min, with an average of 41 min. Using the principles of thematic analysis, four main themes were generated: Theme 1: Network building; Theme 2: Hierarchies and power dynamics; Theme 3: Communication challenges; Theme 4: Career development.

### Theme 1: network building

In this study, almost of all participants recognised that building a network in the conflict and health field was a key output from the R4HC-MENA programme. Having a strong network across institutions within the UK and in other countries could bring a wide range of benefits for the researchers. From the participants’ perspectives, it was believed that this networking could be beneficial to their personal careers, help maintain existing relationships, and expand the existing academic network in this particular field, i.e., conflict and health. In particular, from junior researchers’ accounts, they perceived that this could be a vital opportunity for them to grow their own networks in the field. This active involvement gave the junior researchers a unique opportunity to express their views on the programme. One participant mentioned:

“I really enjoyed meeting other postdocs or research associates. I think that is something that's so clear – strength that's coming out of the projects – and I'm still in touch with various the more junior people on the project, and I'm writing a paper with one. So, so that was real and I guess maybe that was also part of the purpose, was building these networks.” (R01)

Meanwhile, both senior and junior researchers felt that active involvement of researchers in different countries who have similar research interests could help international collaboration in many ways. For example, although some researchers were well experienced in conducting and managing study projects, there was still a need for understanding the research contexts. Some participants felt that this strong multidisciplinary network could improve mutual understanding and establish foundational relationships for the programme. Also, this could then lead to a successful programme and productive outputs from it. One senior researcher described this as below:

“Well, I think we’'ve had a superb team. The team. Oh yeah. Syncing together was a big advantage because we came from different disciplines at the same time. We were all engaged in thinking things out. And I think the beauty of the project has been that team that was built. Over time, I think it was the great team. One of the best I've ever had, really. So that team, ideas, and the way in which these different ideas seeped into what we do and helped us rethink, rework, reconsider and get closer to each other conceptually.” (R04)

Critically, they also stressed that this networking, which was required t o ensure the success of international collaborative studies, could bring not just short-term but also long-term benefits for the researchers in the programme. Some participants pointed out that building a network could contribute to the development of future studies across different workstreams in different countries. However, some also raised a concern about how to maintain this network on a long-term basis. One participant explained:

“You know, my concern is, if I were to step back now, I don't think the network would continue in any shape or form. And I don't know how to articulate that any better way. It's a feeling that I’m the glue, you know. There are one or two people that hold things together. But once you take that out, it does this and dissipates again and everybody goes into their own silos. So, I don't feel that the inertia and the tendency to dissipate is out of the system at all. You know, if I don't continue to put more energy into this, then people will stop working together.” (R03)

### Theme 2: hierarchies and power dynamics

Overwhelmingly, participants from both researchers and stakeholders expressed concerns about the power hierarchy in the research environments in the UK and in the partner countries. Particularly from the perspectives of researchers who self-identified as junior or earlier career researchers, there was a strong sense that there was a power imbalance between senior and junior researchers in the programme. Many participants believed that senior researchers held more powerful positions in the programme. Furthermore, some of the junior researchers felt that they were not able to speak up, and that they often played a relatively silent role in encounters in the field with people from senior levels. For example, one junior researcher reported:

“You know, there's a power dynamic that make it difficult to raise those things if you're a junior scholar.” (R01)

In the participants’ opinion, the power imbalance issue was compounded and more serious in the partner countries. Stakeholders in the UK also identified the power imbalance issue on the ground in the partner countries. They gave some examples to illustrate how early-career researchers did not receive proper credit for published papers or were not acknowledged for the work which they had done for the programme. The members of the R4HC-MENA programme had been aware of this issue and had taken actions to address it. However, from some participants’ views, although some efforts had been made, it seems the issue was not resolved. One participant mentioned that there was ongoing discussion on the issue which had been reported to the programme’s Executive Board meetings. This was illustrated by one participant in the example below:

“Our expectation for the project, for the programme, is that everyone who makes a substantive research contribution to a paper or publication is adequately credited and appropriately credited. It's an important example because this is a UK expectation. And we needed to set that against being sensitive, as I've said, to the institutional norms and expectations for each of our partners in the region. On the one hand, whilst we were very clear about [how] this was an aspiration for the project in terms of equity – of fairness – in publishing credit, we needed to do that in a forceful way, but also in a way that was cognisant of insensitivity to the norms and expectations of our partners. This is an issue that we return to again and again during our Executive Board meetings.” (R07)

Based on the nature of power hierarchy and academic status, some participants also perceived that leadership by senior researchers could therefore be key to deciding whether researchers were able to achieve the goals/objectives of this international collaborative project. Some participants, for example, explained how senior researchers’ leadership styles influenced the project in negative or positive ways. Among the positive examples, the participants described some of the senior researchers as exhibiting “good leadership” when they were very supportive of junior researchers and encouraged them to develop and deliver presentations themselves during the project. Furthermore, their professional experience and management skills in large projects and in particular conflict settings were also seen as critical characteristics of being a “good leader”. For example:

“Under XXX’s leadership…… Ensuring that all of our colleagues across genders, different levels of colleagues as much as possible, had a voice in the group. So, for example, XXX was very clear from the outset that our Executive Board meetings would be, could be and should be attended by all project staff, regardless of whether they'd sort of just joined as research associates or whether they're the senior investigator for the institution. Everybody would be able to and invited to attend all of our Executive Board meetings.” (R07)

However, some found that poor leadership, such as having no clear direction or working plans for the field work, could cause huge workload and pressure for the researchers in the workstreams of the project. One participant reflected here:

“But then when it comes to, like, discussing progress or plans with the workstream, people [workstream leads] in the other places we weren't meeting really as much. There were only some emails exchanged, and I thought, like, it should be more. It was not really well organised, or like a schedule for meetings or a plan, or a workplan.” (R08)

Furthermore, a few participants in the interviews raised the subject of hierarchies and power dynamics issues between countries. In some cases, a lack of clarity about the agenda, and who should take a lead and how, may then have resulted in challenges in linking evidence and practice on the ground. Participants believed that the situation was even more complicated in conflict settings and environments than they had expected. One participant explained how the unique conflict environment influenced cooperation within the R4HC MENA programme, and its ability to bring effective influence to bear at policy-making levels.

“Some research didn't go very well because, frankly….we're not pushing the agenda, they [partner country] weren't really taking the leadership. Because, uh, often the research happend, and then the environment [changed], and the conflict and violence escalate so much. So it's been mixed and it's very hard, and also the connection between the research and policy and practice. Very difficult, much more complicated to find out how that changes downstream policy and practice than it is in permissive stable environments.”(R03)

Additionally, gender power imbalance issues had also been highlighted in the conflict and health research environment. Some participants explained that seeing male researchers as authoritative figures led many female researchers to feel that they were not able to become involved in decision making for the programme. In this case, female researchers felt that male researchers were more likely to hold a powerful role in the decision-making process and field work. This was highlighted by a female participant in the following example:

“You know these [gender inequality issues] are very common problems, and were actually really highlighted from that particular institution, which is very male dominated. It would be incredibly disrespectful. I would, you know, say this behaviour is not good, that you need to show some respect, and you should know you should be accountable for your time on this project... I don't think they would have spoken like that to a male professor. They certainly wouldn’t have spoken to a ‘white male academic’ in the way that they spoke to an ethnic minority female academic. I just don't think it's acceptable.” (R06)

### Theme 3: communication challenges

Due to nature of the programme, the importance of effective communication paths for different group, levels, or countries was emphasised in the participants’ interviews. The corollary, ineffective communication, was another key concern raised in the interviews as causing problems for the management of international collaborative projects in the conflict and health research field. International collaborative studies such as the R4HC-MENA programme often involve different workstreams and require people from different institutions and countries to affiliate and collaborate together. The participants in this study believed that multiple research teams and various workstreams should actively engage in, and communicate throughout, the programme. Beyond only individual workstreams, some were also aware of the importance of communication across different workstreams. They felt some lessons could be learnt by working and exchanging information with other workstreams. One participant explained:

“At the beginning, I was hoping to see more linkages between these workstreams. They were, of course, in the executive board meeting, etc. But I think it's still… I felt like everyone is working in [their] own territory. So, this could be also something that can be enhanced through a second cycle of the project.” (R10)

Furthermore, one participant also pointed out the importance of effective communication within a workstream and explained how he/she maintains long-term working relationship with the other researcher:

“We're friends, we know how we work, we know how we think. Brought us together as a team. I'm working with XXX and her/his team since 2012. So that's friendship. That's mutual understanding and that's respect. There is joint and long-time development of joint ways of working. We know each other’s place in this collaboration. So, in that sense, it was smooth and straightforward and actually really enjoyable.” (R04)

However, some participants also reported the issue that institutions not working as a team contributed to weaknesses in managing individual projects. Tensions and faulty communications between the institutions then created work pressure for researchers on the ground, particularly for junior researchers. One researcher explained:

“But XXX [institution] doesn't want to say this direct message to YYY [institution] so that they don't create this tension. So what happened here was that I was in the middle of two institutions… And neither of them would talk and decide on this. But I was caught in the middle and I'll be the one who doesn't even know how to deal with the situation.” (R02)

Participants also proposed potential solutions for ineffective communication challenges in the interviews and emphasised the need for effective communication methods. Some participants felt that more regular meetings would help those involved in the project to communicate well and build relationships within different workstreams. However, opinions on the appropriate size of meetings varied between the interviews. For example, some felt that meetings for all staff and researchers from all levels could enhance mutual understanding across different workstreams:

“For example, in the workstreams, I felt like we could have had more regular meetings.

More meetings with the other teams… And the first time I, like, really interacted with them [other teams] was in Jordan's face-to-face meeting…..With the, all the other people working on a specific workstream and, yeah, could have been more organised.” (R08)

However, some felt that junior researchers did not have sufficient time or opportunities to carry out their work. Therefore, meetings or workshops involving fewer people could be more beneficial for the researchers in the programme. One junior participant reported:

“So I think a little bit more thought about what those meetings were for would have been helpful. I think they weren't necessarily a help. Because then we'd spend a couple weeks before the meetings planning what to say in the meeting rather than actually doing any of the research. So, there's a lot of – kind of duplication of effort. Spending a lot of time talking about what you're doing rather than actually doing it. I think it's probably important to figure out a way of overcoming that.” (R03)

A few participants also made specific reference to the challenging communication methods adopted during the COVID-19 pandemic, especially in humanitarian contexts. Although some benefits of virtual meetings were reported, such as flexibility, the importance of physical interaction between people in physical and face-to-face meetings was also highlighted in the interviews. It seems that such collaborations in humanitarian contexts require more effective communication to ensure smooth workflow. For example:

“Now, actually about learning how we could work together as a group and genuinely co-creating research agendas together. It’s very hard to do on Zoom, I think. And especially thinking back to what I was saying at the beginning about it being a highly politicized group, people and a situation where working that needs face-to-face time. There are issues that make doing research in those [conflict] settings challenging – especially challenging. And I think this that goes particularly true for XX [one partner country]. And you know, the last kind of two years of the project was hamstrung really by a series of events [humanitarian contexts/events] in XX [one partner country] that nobody could have foreseen. So you know, I, again, I think it became very challenging.” (R08)

### Theme 4: career development

Another key theme reported by the participants was how their active involvement and experience in the programme had contributed to their personal/career development, including improving personal skills and advancing along career pathways. In the interviews, participants said that this programme could have fostered their management, leadership, research, and teaching skills for their personal development. The idea that some real experience of field work in conflict and health settings was valuable for building research capacities for the researchers was widely reported in the interviews. One participant said:

“It's widened, in my view, and I brought my skills and knowledge from the project. Also, as you know, that also is capacity building. I'm grateful to study a postgraduate degree [under the project]. That was very good experience as well and improved my career as, you know, in research and XX [subject] as a whole.” (R09)

The majority of the participants also recognised the academic publications in conflict and health as vital outputs and proof of all the workstreams through the programme. Again, these valuable publications were seen as key milestones for their personal career development. One participant mentioned:

“I mean, the networks again. So, so the people from my workstream, I’m still absolutely part of [that network]. And we produced and created a couple of decent papers – that were amazing papers – from the projects [R4HC MENA]. So I think those are the kind of the main [outputs].” (R01)

Beyond this, more than half of the participants in the interviews highlighted that a mentor support system, where senior researchers mentored junior researchers in the programme, is a key foundation for the careers and personal development of the latter. It was believed that this mentoring dynamic in the programme could be helpful for the development both of those being mentored and of the mentors themselves at the same time. Also, the participants believed that this had increased the motivation, confidence, and independence in conflict and health studies of junior researchers. One example:

“I think that's a great way for the project to really support early career researchers and people to develop their – their own skills. Yeah, and to be taught by key persons and, you know, around the world about this kind of research was very, very useful for me.” (R09)

In this study, a few participants felt that more expert training for researchers on conducting research was needed. It was widely reported by the junior participants that they had insufficient training to put research theories into practice. Interestingly, this drew attention from the junior researchers only. In their accounts, they emphasised that they needed more knowledge of particular research techniques to achieve the objectives of the R4HC-MENA programme. One participant described his/her experience as follows:

“We didn't have any formal training or anything like that [qualitative study training]. It was just “Go out and do it.” Again, independence can be good. But a little bit more direction, I think, would have helped me. I think the other RA [research associate] felt that as well.” (R01)

Another issue stated by the junior participants, such as research assistants or research associates, was that they only held fixed-term contract positions in the programme. Therefore, they felt job uncertainty and had no long-term security because of their fixed-term status. Some participants also mentioned that being precariously employed negatively influenced their willingness to be involved in the programme. One example:

“There is a challenging part for me: rapid turnover… We had the rapid turnover in the group as well. It's a massive challenge. And I think that's also part of, you know, working in academia. Yeah, because this is a temporary job, you know, the project would be finished one day, you need a better job.” (R11)

In particular, unstable situations and environment in the conflict settings, such as governance failures, displacement of the population, and breakdown of health and social services, have been recognised as barriers in international collaboration projects. Uncertainty in the countries force the populations to leave their own countries. This often influences all aspects of daily life in the field. One participant explained that the issue also dramatically influenced their feelings of job security as academics in the field.

“And it's something that happened a lot in XX [one partner country]. In specific, because of the situation. So because of the political instability, the economic decline and a lot of things that happened, [it] made a lot of us just choose to leave the country… Uh, how this impacted the work, of course. And I think that this is one of the major issues of, or for, R4HC-MENA, and it's not, like, specific for R4HC only but actually all of academia. It [has strong impact] especially for those early career researchers because they don't have some sort of, like, job security.” (R02)

## Discussion

Overall, four main themes related to challenges and key outputs of the programme were identified: Network building; Hierarchies and power dynamics; Communication challenges; Career development. Although benefits of the international collaboration were discussed in the interviews, the instability in conflict settings was also pointed out by the participants. It was believed that this makes international collaborative projects even more complex and challenging.

Both this study and previous research described in the literature found that network building and career development could be key benefits for researchers working on international collaborative projects [38–40]. One of the goals of the R4HC-MENA programme was to encourage members and researchers from partner countries to improve their academic career development or pursue higher education. This, then, echoed the literature on the benefits of international collaborative projects [38], and might explain why, although some challenges emerged in such projects, the researchers still found it to be of importance and value to be involved in them. Similarly, one recent publication also found that international research consortia can strengthen organisations’ research capacity and provide an inclusive environment for academics [40]. This might also be vital for encouraging researchers, particularly junior/early career researchers, to actively engage in international collaborative projects. Given their benefits, how to sustain these networks could also be vital in the future. The Centre for Conflict & Health Research (CCHR) at King’s College London was established following the completion of the R4HC MENA programme. This centre provides a unique opportunity to extend R4HC MENA partnership further, bringing together members from across King’s College London and King’s Health Partners with global collaborations. Although further funds have been secured (NIHR funded £4 m Research for Health Systems Strengthening in Syria (R4HSSS) programme) and some potential funding opportunities have been explored, concerns about substantial funds for the further collaboration were also raised in the interviews. Some actions might need to be taken for long-term collaboration and the maintenance of the network.

However, other challenges were also discussed in the study: power imbalance and ineffective communication for the R4HC MENA programme. Evidence from this study suggested that the researchers experienced or witnessed power imbalance during international collaboration for a project on conflict and health research. Similarly, a strong sense of power imbalance is still commonly found in the research environment in the literature [41, 42]. Overwhelmingly, the particular issue that junior and female researchers are more likely to keep silent during project decision making processes was widely reported by the researchers both in the UK and in the partner countries in this study; this also is reflected in the current literature [41–44]. A male-dominated environment is still widespread in research institutions in many countries, including the USA [45, 46], the UK [47], the Middle East countries [48], African countries [44], and South Asian countries [43]. This might explain why power imbalances were observed in the study. This again echoes what participants in this study found: that it is even more complicated in the conflict setting and humanitarian contexts than in relatively stable environments. The issue of the effect of power imbalance related to researcher status and gender still needs further action to make equality possible in this field.

Meanwhile, the findings of this study showed another challenge in the international collaboration programme: ineffective communication. Ineffective and unclear communication between researchers at different levels was found in this study to have negatively influenced the collaborative programme, and is also reported in the literature [49]. With ineffective and unclear communication channels, members could fail to grasp the exact problem in a timely manner, share information, and accurately interpret information. For international projects, differences in culture and language could worsen the communication issues between host and partner countries [49]. Additionally, particular challenges in the conflict setting and humanitarian contexts during COVID-19 were highlighted in the study. This, then, needs more attention to develop further effective strategies to overcome communication barriers and improve communication paths for international collaborative projects.

## Limitations of the study

Although this study has provided an insight into how researchers and stakeholders view international collaboration on a programme on conflict and health research, there are limitations to this study. The findings should be carefully considered because of the small number of respondents (n = 12), including three participants who did not complete the background information questionnaire. It was therefore difficult to provide a full picture of the sample for readers. In addition, the population was only recruited from one international collaborative research programme (R4HC-MENA). Also, more than half of the participants were based in the UK (n = 8), and only four participants were based in other countries (three women and one man, 2 researchers and two stakeholders). The representation of the partner countries should then carefully be taken into account. Despite qualitative studies not being intended to be generalisable, more diverse samples could be considered for subsequent studies to ensure that the data is transferable to other contexts and settings. However, this data provides insights that could inform further studies on the internal dynamics, benefits and challenges of international research programmes for early career participants.

## Conclusion

This study provided a unique opportunity for the researchers to explain their perspectives on international collaboration in a major international programme of research on conflict and health. It also showed that building networks, supporting personal development, and career progression, were perceived to be important benefits for researchers at all levels in the programme. Although there were differences in power hierarchies in different institutions and countries, all participants expected a culture with a better power balance in the conflict and health research enviroment. Some key challenges were generated in this study. Given the importance of these, more attention should be paid to offering appropriate support and effective training for researchers involved in international projects. These challenges of conducting and being involved in international collaboration identified by this study should be carefully considered in the field. Some efforts could be made to structure effectively international collaborations focused on conflict and health research, or more generally for humanitarian health research, such as joint communication platforms, mutural understanding, and cultural adaptation (regularly reviewing and changing the structure of a collaborative programme). The findings could also then be an important foundation for further developing effective strategies to tackle the challenge of power imbalance and ineffective communication in internatioanl collaborative partnerships.

## Data Availability

The interveiw data used in this study are not publicly available due to integrity statements in the ethical approvals.

## Abbreviations

R4HC-MENA: Research for Health Conflict—Middle East and North Africa
WLHC: Women Leaders in Health and Conflict
FCDO: Foreign, Commonwealth & Development Office
